# Supplementary material for: The effect of access to water, sanitation and handwashing facilities on child growth indicators: Evidence from the Ethiopia Demographic and Health Survey 2016
Source: PLoS One. 2020 Sep 22;15(9):e0239313. doi: 10.1371/journal.pone.0239313 (PMC7508389; doi:10.1371/journal.pone.0239313)
Supplement: S1 Table — (DOCX) [file pone.0239313.s001.docx]

**S1 Table.** Univariable and multivariable analyses results of stunting for all confounding variables, EDHS 2016 (weighted n = 9588)

| **Variables** | **Stunting** | | **Model 0** | | **Model 1** | | **Model 2** | | **Model 3** | | **Model 4** | | **Model 5** | |
| --- | --- | --- | --- | --- | --- | --- | --- | --- | --- | --- | --- | --- | --- | --- |
|  | **No** | **Yes** | **COR (95%CI)** | **P value** | **AOR (95%CI)** | **P value** | **AOR (95%CI)** | **P value** | **AOR (95%CI)** | **P value** | **AOR (95%CI)** | **P value** | **AOR (95%CI)** | **P value** |
| **Water facility** |  |  |  |  |  |  |  |  |  |  |  |  |  |  |
| Improved | 3361 | 1958 | 0.86 (0.74, 0.99) | 0.002 |  |  | 1.06 (0.89, 1.26) | 0.526 |  |  |  |  |  |  |
| Unimproved | 2474 | 1684 | Ref |  |  |  | Ref |  |  |  |  |  |  |  |
| **Sanitation facility** |  |  |  |  |  |  |  |  |  |  |  |  |  |  |
| Improved | 673 | 250 | 0.57 (0.44, 0.73) | < 0.001 |  |  |  |  | 0.89 (0.66, 1.21) | 0.453 |  |  |  |  |
| Unimproved | 5162 | 3392 | Ref |  |  |  |  |  | Ref |  |  |  |  |  |
| **Handwashing facility** |  |  |  |  |  |  |  |  |  |  |  |  |  |  |
| Improved | 3148 | 1877 | 0.91 (0.79, 1.04) | 0.040 |  |  |  |  |  |  | 0.98 (0.84, 1.14) | 0.755 |  |  |
| Unimproved | 2688 | 1764 | Ref |  |  |  |  |  |  |  | Ref |  |  |  |
| **WASH facilities** |  |  |  |  |  |  |  |  |  |  |  |  |  |  |
| Improved | 420 | 109 | 0.40 (0.29, 0.55) | < 0.001 |  |  |  |  |  |  |  |  | 0.67 (0.45, 0.98) | 0.040 |
| Unimproved | 5416 | 3533 | Ref |  |  |  |  |  |  |  |  |  | Ref |  |
| **Region** |  |  |  |  |  |  |  |  |  |  |  |  |  |  |
| Addis Ababa | 180 | 31 | Ref |  | Ref |  | Ref |  | Ref |  | Ref |  | Ref |  |
| Tigray | 397 | 252 | 3.69 (2.60, 5.24) | < 0.001 | 1.94 (1.22, 3.09) | 0.005 | 2.12 (1.36, 3.31) | 0.001 | 2.03 (1.29, 3.19) | 0.002 | 2.13 (1.36, 3.31) | 0.001 | 1.76 (1.12, 2.78) | 0.015 |
| Afar | 54 | 37 | 3.98 (2.78, 5.71) | < 0.001 | 1.20 (0.71, 2.01) | 0.496 | 1.37 (0.83, 2.26) | 0.219 | 1.30 (0.78, 2.17) | 0.310 | 1.36 (0.82, 2.25) | 0.229 | 1.13 (0.67, 1.92) | 0.639 |
| Amhara | 992 | 886 | 5.19 (3.64, 7.40) | < 0.001 | 2.50 (1.57, 3.99) | < 0.001 | 2.89 (1.85, 4.53) | <0.001 | 2.71 (1.71, 4.29) | < 0.001 | 2.89 (1.84, 4.52) | < 0.001 | 2.34 (1.48, 3.72) | < 0.001 |
| Oromia | 2680 | 1524 | 3.31 (2.33, 4.68) | < 0.001 | 1.41 (0.87, 2.28) | 0.167 | 1.55 (0.98, 2.44) | 0.060 | 1.46 (0.92, 2.31) | 0.111 | 1.54 (0.97, 2.42) | 0.065 | 1.26 (0.78, 2.03) | 0.351 |
| Somali | 290 | 107 | 2.15 (1.49, 3.10) | < 0.001 | 0.66 (0.40, 1.10) | 0.107 | 0.74 (0.46, 1.22) | 0.238 | 0.72 (0.44, 1.18) | 0.188 | 0.73 (0.45, 1.20) | 0.222 | 0.62 (0.37, 1.02) | 0.057 |
| Benishangul-Gumuz | 58 | 43 | 4.36 (3.04, 6.25) | < 0.001 | 2.09 (1.27, 3.42) | 0.004 | 2.18 (1.35, 3.51) | 0.001 | 2.08 (1.28, 3.39) | 0.003 | 2.21 (1.37, 3.55) | 0.001 | 1.80 (1.11, 2.94) | 0.018 |
| SNNP | 1204 | 774 | 3.74 (2.61, 5.34) | < 0.001 | 1.82 (1.14, 2.93) | 0.013 | 1.95 (1.24, 3.08) | 0.004 | 1.84 (1.15, 2.93) | 0.011 | 1.94 (1.23, 3.05) | 0.005 | 1.59 (1.00, 2.53) | 0.051 |
| Gambela | 17 | 5 | 1.77 (1.20, 2.61) | 0.004 | 0.85 (0.53, 1.38) | 0.518 | 0.90 (0.56, 1.44) | 0.662 | 0.86 (0.53, 1.39) | 0.535 | 0.91 (0.57, 1.45) | 0.680 | 0.75 (0.46, 1.21) | 0.237 |
| Harari | 14 | 6 | 2.72 (1.83, 4.04) | < 0.001 | 1.42 (0.86, 2.35) | 0.172 | 1.55 (0.95, 2.51) | 0.077 | 1.48 (0.91, 2.42) | 0.115 | 1.53 (0.94, 2.50) | 0.089 | 1.27 (0.77, 2.10) | 0.355 |
| Dire Dawa | 21 | 15 | 4.08 (2.71, 6.14) | < 0.001 | 1.93 (1.21, 3.07) | 0.006 | 2.12 (1.34, 3.36) | 0.001 | 2.08 (1.31, 3.30) | 0.002 | 2.11 (1.33, 3.35) | 0.002 | 1.83 (1.19, 2.98) | 0.010 |
| **Wealth index** |  |  |  |  |  |  |  |  |  |  |  |  |  |  |
| Poorest | 1214 | 997 | Ref |  | Ref |  | Ref |  | Ref |  | Ref |  | Ref |  |
| Poorer | 1281 | 969 | 0.92 (0.75, 1.13) | 0.432 | 0.94 (0.74, 1.22) | 0.679 | 0.91 (0.71, 1.17) | 0.469 | 0.92 (0.72, 1.18) | 0.521 | 0.92 (0.72, 1.18) | 0.511 | 0.92 (0.72, 1.18) | 0.517 |
| Middle | 1257 | 761 | 0.74 (0.59, 0.93) | 0.009 | 0.77 (0.59, 1.00) | 0.052 | 0.73 (0.56, 0.94) | 0.017 | 0.74 (0.57, 0.96) | 0.026 | 0.74 (0.57, 0.96) | 0.025 | 0.74 (0.57, 0.96) | 0.025 |
| Richer | 1137 | 605 | 0.65 (0.53, 0.80) | < 0.001 | 0.72 (0.55, 0.94) | 0.014 | 0.66 (0.52, 0.85) | 0.001 | 0.68 (0.54, 0.87) | 0.002 | 0.68 (0.53, 0.87) | 0.003 | 0.69 (0.54, 0.87) | 0.002 |
| Richest | 1018 | 349 | 0.42 (0.33, 0.53) | < 0.001 | 0.63 (0.45, 0.88) | 0.007 | 0.53 (0.39, 0.71) | < 0.001 | 0.56 (0.41, 0.77) | < 0.001 | 0.55 (0.41, 0.74) | < 0.001 | 0.59 (0.44, 0.80) | 0.001 |
| **Sex of the child** |  |  |  |  |  |  |  |  |  |  |  |  |  |  |
| Male | 2876 | 2017 | Ref |  | Ref |  | Ref |  | Ref |  | Ref |  | Ref |  |
| Female | 3031 | 1663 | 0.78 (0.69, 0.89) | < 0.001 | 0.77 (0.66, 0.88) | < 0.001 | 0.77 (0.67, 0.89) | <0.001 | 0.77 (0.67, 0.89) | < 0.001 | 0.77 (0.67, 0.89) | < 0.001 | 0.77 (0.67, 0.89) | 0.001 |
| **Age of child (months)** |  |  |  |  |  |  |  |  |  |  |  |  |  |  |
| 0-11 | 1755 | 351 | Ref |  | Ref |  | Ref |  | Ref |  | Ref |  | Ref |  |
| 12-23 | 1127 | 780 | 3.46 (2.65, 4.50) | < 0.001 | 3.82 (2.74, 5.34) | < 0.001 | 3.97 (2.84, 5.57) | < 0.001 | 3.98 (2.85, 5.58) | < 0.001 | 3.98 (2.84, 5.57) | < 0.001 | 4.01 (2.86, 5.62) | < 0.001 |
| 24-35 | 927 | 871 | 4.69 (3.66, 6.02) | < 0.001 | 6.30 (4.49, 8.83) | < 0.001 | 6.62 (4.69, 9.34) | < 0.001 | 6.65 (4.71, 9.39) | < 0.001 | 6.63 (4.70, 9.35) | < 0.001 | 6.72 (4.75, 9.50) | < 0.001 |
| 36-47 | 971 | 860 | 4.43 (3.50, 5.59) | < 0.001 | 6.77 (4.71, 9.73) | < 0.001 | 7.32 (5.05, 10.61) | < 0.001 | 7.38 (5.09, 10.7) | < 0.001 | 7.34 (5.07, 10.62) | < 0.001 | 7.43 (5.12, 10.8) | < 0.001 |
| 48-59 | 1126 | 819 | 3.64 (2.81, 4.70) | < 0.001 | 6.19 (4.25, 9.04) | < 0.001 | 6.79 (4.59, 10.03) | < 0.001 | 6.84 (4.63, 10.1) | < 0.001 | 6.81 (4.61, 10.05) | < 0.001 | 6.92 (4.67, 10.2) | < 0.001 |
| **Child anaemia status** |  |  |  |  |  |  |  |  |  |  |  |  |  |  |
| Sever | 119 | 133 | Ref |  | Ref |  | Ref |  | Ref |  | Ref |  | Ref |  |
| Moderate | 2538 | 1991 | 0.70 (0.47, 1.05) | 0.08 | 0.77 (0.50, 1.19) | 0.233 | 0.76 (0.49, 1.18) | 0.219 | 0.75 (0.48, 1.18) | 0.213 | 0.76 (0.49, 1.17) | 0.212 | 0.76 (0.49, 1.18) | 0.221 |
| Not anaemic | 2235 | 1330 | 0.53 (0.36, 0.79) | 0.002 | 0.46 (0.30, 0.72) | 0.001 | 0.46 (0.29, 0.71) | 0.001 | 0.45 (0.29, 0.71) | 0.001 | 0.45 (0.29, 0.71) | 0.001 | 0.45 (0.29, 0.71) | 0.001 |
| **Childbirth order** | 5907 | 3681 | 1.03 (1.00, 1.06) | 0.015 | 1.08 (1.02, 1.14) | 0.008 | 1.07 (1.01, 1.14) | 0.016 | 1.07 (1.01, 1.13) | 0.017 | 1.07 (1.01, 1.13) | 0.017 | 1.07 (1.01, 1.13) | 0.021 |
| **Size of child at birth** |  |  |  |  |  |  |  |  |  |  |  |  |  |  |
| Larger than average | 1954 | 1044 | Ref |  | Ref |  | Ref |  | Ref |  | Ref |  | Ref |  |
| Average | 2577 | 1526 | 1.11 (0.96, 1.28) | 0.151 | 1.15 (0.98, 1.34) | 0.091 | 1.16 (0.99, 1.35) | 0.07 | 1.16 (0.99, 1.35) | 0.07 | 1.15 (0.99, 1.35) | 0.072 | 1.15 (0.98, 1.35) | 0.078 |
| Smaller than average | 1376 | 1111 | 1.51 (1.27, 1.80) | < 0.001 | 1.52 (1.26, 1.84) | < 0.001 | 1.58 (1.31, 1.90) | < 0.001 | 1.58 (1.31, 1.90) | < 0.001 | 1.58 (1.31, 1.90) | < 0.001 | 1.57 (1.30, 1.89) | < 0.001 |
| **Breastfeeding status** |  |  |  |  |  |  |  |  |  |  |  |  |  |  |
| Never breastfed | 199 | 146 | Ref |  | Ref |  | Ref |  | Ref |  | Ref |  | Ref |  |
| Still breastfeeding | 2982 | 1571 | 0.72 (0.50-1.04) | 0.084 | 1.21 (0.80, 1.84) | 0.364 | 1.20 (0.78, 1.83) | 0.401 | 1.21 (0.80, 1.83) | 0.373 | 1.21 (0.80, 1.84) | 0.366 | 1.19 (0.78-1.81) | 0.431 |
| Ever breastfed | 2726 | 1964 | 0.98 (0.69-1.41) | 0.930 | 0.79 (0.53, 1.16) | 0.228 | 0.77 (0.52, 1.15) | 0.199 | 0.77 (0.52, 1.15) | 0.199 | 0.30 (0.03, 3.34) | 0.209 | 0.76 (0.51-1.13) | 0.178 |
| **Diarrhoea in 2 weeks** |  |  |  |  |  |  |  |  |  |  |  |  |  |  |
| Yes | 681 | 469 | 1.12 (0.90, 1.39) | 0.143 | 1.15 (0.91, 1.46) | 0.334 | 1.15 (0.91, 1.45) | 0.340 | 1.15 (0.91, 1.45) | 0.345 | 1.15 (0.91, 1.45) | 0.339 | 1.15 (0.91, 1.46) | 0.348 |
| No | 5210 | 3209 | Ref |  | Ref |  | Ref |  | Ref |  | Ref |  | Ref |  |
| **Maternal age (years)** | 5907 | 3681 | 1.01 (0.99, 1.02) | 0.088 | 0.97 (0.95, 0.99) | 0.002 | 0.97 (0.95, 0.99) | 0.006 | 0.97 (0.95, 0.99) | 0.006 | 0.97 (0.95, 0.99) | 0.006 | 0.97 (0.95, 0.99) | 0.007 |
| **Birth interval** |  |  |  |  |  |  |  |  |  |  |  |  |  |  |
| < 24 months | 901 | 707 | 1.30 (1.09, 1.56) | < 0.001 | 1.23 (1.01, 1.49) | 0.041 | 1.18 (0.98, 1.43) | 0.217 | 1.10 (0.86, 1.40) | 0.211 | 1.18 (0.98, 1.43) | 0.211 | 1.18 (0.98, 1.43) | 0.211 |
| >= 24 months | 3882 | 2341 | Ref |  | Ref |  | Ref |  | Ref |  | Ref |  | Ref |  |
| **Number of ANC visits** |  |  |  |  |  |  |  |  |  |  |  |  |  |  |
| < 4 | 2810 | 1787 | 1.44 (1.23, 1.69) | < 0.001 | 1.28 (1.08, 1.52) | 0.016 | 1.29 (1.09, 1.53) | 0.013 | 1.29 (1.09, 1.53) | 0.014 | 1.29 (1.09, 1.52) | 0.014 | 1.28 (1.08, 1.52) | 0.016 |
| >= 4 | 1498 | 663 | Ref |  | Ref |  | Ref |  | Ref |  | Ref |  | Ref |  |
| **Minimum food groups consumed in 24hrs** |  |  |  |  |  |  |  |  |  |  |  |  |  |  |
| < 4 food groups | 1845 | 1061 | 2.11 (1.13, 3.95) | < 0.001 | 2.31 (1.13, 4.74) | 0.022 | 2.28 (1.10, 4.75) | 0.027 | 2.27 (1.10, 4.66) | 0.026 | 2.26 (1.10, 4.66) | 0.027 | 2.27 (1.11, 4.66) | 0.028 |
| >= 4 food groups | 95 | 26 | Ref |  | Ref |  | Ref |  | Ref |  | Ref |  | Ref |  |

AOR, adjusted odds ratio; ANC, antenatal care; COR, crude odds ratio; Ref, reference group; SNNP, southern nations, nationalities and people; WASH, water, sanitation and handwashing; Model 0, results from unadjusted univariable analysis; Model 1, adjusted for all variables with p value < 0.25 in model 0; Model 2, adjusted for water plus all variables with p value < 0.05 in Model 1; Model 3, adjusted for sanitation plus all variables with p value < 0.05 in Model 1; Model 4, adjusted for handwashing plus all variables with p value < 0.05 in Model 1; Model 5, adjusted for combined WASH facilities plus all variables with p value < 0.05 in Model 1.
