# Supplementary material for: The effect of access to water, sanitation and handwashing facilities on child growth indicators: Evidence from the Ethiopia Demographic and Health Survey 2016
Source: PLoS One. 2020 Sep 22;15(9):e0239313. doi: 10.1371/journal.pone.0239313 (PMC7508389; doi:10.1371/journal.pone.0239313)
Supplement: S2 Table — (DOCX) [file pone.0239313.s002.docx]

**S2 Table.** Univariable and multivariable analyses results of underweight for all confounding variables, EDHS 2016 (weighted n = 9752)

| **Variables** | **Underweight** | | **Model 0** | | **Model 1** | | **Model 2** | | **Model 3** | | **Model 4** | | **Model 5** | |
| --- | --- | --- | --- | --- | --- | --- | --- | --- | --- | --- | --- | --- | --- | --- |
|  | **No** | **Yes** | **COR (95%CI)** | **P value** | **AOR (95%CI)** | **P value** | **AOR (95%CI)** | **P value** | **AOR (95%CI)** | **P value** | **AOR (95%CI)** | **P value** | **AOR (95%CI)** | **P value** |
| **Water facility** |  |  |  |  |  |  |  |  |  |  |  |  |  |  |
| Improved | 4196 | 1234 | 0.88 (0.74, 1.04) | 0.127 |  |  | 1.14 (0.95,1.37) | 0.152 |  |  |  |  |  |  |
| Unimproved | 3155 | 1056 | Re |  |  |  | Ref |  |  |  |  |  |  |  |
| **Sanitation facility** |  |  |  |  |  |  |  |  |  |  |  |  |  |  |
| Improved | 775 | 152 | 0.60 (0.43, 0.84) | < 0.001 |  |  |  |  | 0.95 (0.66, 1.37) | 0.785 |  |  |  |  |
| Unimproved | 6575 | 2139 | Ref |  |  |  |  |  | Ref |  |  |  |  |  |
| **Handwashing facility** |  |  |  |  |  |  |  |  |  |  |  |  |  |  |
| Improved | 3998 | 1099 | 0.77 (0.67, 0.90) | < 0.001 |  |  |  |  |  |  | 0.83 (0.71, 0.98) | 0.032 |  |  |
| Unimproved | 3353 | 1192 | Ref |  |  |  |  |  |  |  | Ref |  |  |  |
| **WASH facilities** |  |  |  |  |  |  |  |  |  |  |  |  |  |  |
| Improved | 473 | 60 | 0.39 (0.26, 0.61) | < 0.001 |  |  |  |  |  |  |  |  | 0.78 (0.48, 1.27) | 0.313 |
| Unimproved | 6878 | 2230 | Ref |  |  |  |  |  |  |  |  |  | Ref |  |
| **Region** |  |  |  |  |  |  |  |  |  |  |  |  |  |  |
| Addis Ababa | 202 | 11 | Ref |  | Ref |  | Ref |  | Ref |  | Ref |  | Ref |  |
| Tigray | 509 | 149 | 5.36 (2.77, 10.38) | < 0.001 | 2.41 (1.13, 5.10) | 0.022 | 2.61 (1.26, 5.41) | 0.01 | 2.61 (1.23, 5.51) | 0.012 | 2.55 (1.23, 5.31) | 0.012 | 2.37 (1.11, 5.09) | 0.026 |
| Afar | 60 | 34 | 10.41 (5.30, 20.5) | < 0.001 | 2.65 (1.19, 5.88) | 0.017 | 3.11 (1.44, 6.73) | 0.004 | 3.06 (1.40, 6.72) | 0.005 | 2.92 (1.35, 6.34) | 0.007 | 2.80 (1.25, 6.25) | 0.012 |
| Amhara | 1344 | 552 | 7.53 (3.88, 14.64) | < 0.001 | 3.17 (1.47, 6.83) | 0.003 | 3.71 (1.77, 7.79) | 0.001 | 3.59 (1.67, 7.72) | 0.001 | 3.75 (1.78, 7.91) | 0.001 | 3.27 (1.50, 7.10) | 0.003 |
| Oromia | 3317 | 963 | 5.33 (2.76, 10.29) | < 0.001 | 2.14 (0.99, 4.66) | 0.054 | 2.53 (1.20, 5.33) | 0.015 | 2.45 (1.14, 5.27) | 0.021 | 2.40 (1.14, 5.07) | 0.022 | 2.23 (1.01, 4.91) | 0.047 |
| Somali | 293 | 114 | 7.14 (3.65, 13.97) | < 0.001 | 2.18 (0.99, 4.84) | 0.055 | 2.65 (1.24, 5.64) | 0.018 | 2.59 (1.21, 5.56) | 0.015 | 2.50 (1.17, 5.34) | 0.018 | 2.35 (1.07, 5.16) | 0.033 |
| Benishangul-Gumuz | 68 | 36 | 9.65 (4.85, 19.21) | < 0.001 | 4.87 (2.21, 10.7) | < 0.001 | 5.44 (2.54, 11.7) | < 0.001 | 5.49 (2.50, 12.1) | < 0.001 | 5.53 (2.57, 11.88) | < 0.001 | 4.99 (2.24, 11.1) | < 0.001 |
| SNNP | 1584 | 438 | 5.08 (2.60, 9.93) | < 0.001 | 2.40 (1.11, 5.16) | 0.026 | 2.73 (1.30, 5.73) | 0.010 | 2.65 (1.24, 5.67) | 0.012 | 2.62 (1.25, 5.50) | 0.011 | 2.41 (1.11, 5.23) | 0.026 |
| Gambela | 18 | 4 | 4.11 (2.03, 8.30) | < 0.001 | 1.96 (0.89, 4.31) | 0.093 | 2.05 (0.96, 4.39) | 0.064 | 2.05 (0.94, 4.48) | 0.071 | 2.04 (0.95, 4.37) | 0.068 | 1.87 (0.85, 4.14) | 0.122 |
| Harari | 16 | 4 | 4.63 (2.33, 9.21) | < 0.001 | 2.40 (1.10, 5.24) | 0.028 | 2.69 (1.27, 5.72) | 0.010 | 2.63 (1.22, 5.67) | 0.013 | 2.45 (1.15, 5.23) | 0.020 | 2.38 (1.08, 5.24) | 0.031 |
| Dire Dawa | 27 | 10 | 7.01 (3.51, 14.02) | < 0.001 | 2.95 (1.37, 6.34) | 0.006 | 3.29 (1.57, 6.88) | 0.002 | 3.29 (1.56, 6.94) | 0.002 | 3.08 (1.47, 6.48) | 0.003 | 3.04 (1.42, 6.50) | 0.004 |
| **Maternal education** |  |  |  |  |  |  |  |  |  |  |  |  |  |  |
| Has education | 2793 | 558 | Ref |  | Ref |  | Ref |  | Ref |  | Ref |  | Ref |  |
| Has no education | 4645 | 1756 | 1.89 (1.58, 2.27) | < 0.001 | 1.31 (1.06, 1.62) | 0.014 | 1.40 (1.15, 1.72) | 0.001 | 1.40 (1.15, 1.72) | 0.001 | 1.40 (1.14, 1.71) | 0.001 | 1.40 (1.15, 1.71) | 0.001 |
| **Wealth index** |  |  |  |  |  |  |  |  |  |  |  |  |  |  |
| Poorest | 1574 | 705 | Ref |  | Ref |  | Ref |  | Ref |  | Ref |  | Ref |  |
| Poorer | 1658 | 625 | 0.84 (0.69, 1.03) | 0.096 | 0.97 (0.75, 1.25) | 0.815 | 0.93 (0.73, 1.19) | 0.550 | 0.94 (0.74, 1.21) | 0.640 | 0.95 (0.75, 1.22) | 0.703 | 0.94 (0.74, 1.21) | 0.638 |
| Middle | 1562 | 472 | 0.68 (0.53, 0.85) | 0.001 | 0.80 (0.61, 1.05) | 0.112 | 0.73 (0.56, 0.96) | 0.023 | 0.76 (0.58, 0.99) | 0.045 | 0.77 (0.59, 1.01) | 0.055 | 0.76 (0.58, 0.99) | 0.045 |
| Richer | 1461 | 304 | 0.46 (0.36, 0.59) | < 0.001 | 0.60 (0.44, 0.81) | 0.001 | 0.53 (0.40, 0.71) | < 0.001 | 0.56 (0.42, 0.74) | < 0.0001 | 0.57 (0.43, 0.76) | < 0.001 | 0.56 (0.42, 0.74) | < 0.001 |
| Richest | 1183 | 208 | 0.39 (0.29, 0.53) | < 0.001 | 0.85 (0.56, 1.29) | 0.438 | 0.66 (0.46, 0.93) | 0.018 | 0.71 (0.50, 1.01) | 0.057 | 0.74 (0.53, 1.06) | 0.098 | 0.73 (0.52, 1.04) | 0.078 |
| **Sex of the child** |  |  |  |  |  |  |  |  |  |  |  |  |  |  |
| Male | 3736 | 1267 | Ref |  | Ref |  | Ref |  | Ref |  | Ref |  | Ref |  |
| Female | 3702 | 1048 | 0.84 (0.73, 0.96) | < 0.001 | 0.81 (0.70, 0.94) | 0.004 | 0.81 (0.70, 0.94) | 0.005 | 0.81 (0.70, 0.93) | 0.004 | 0.80 (0.69, 0.93) | 0.003 | 0.81 (0.70, 0.93) | 0.004 |
| **Age of child (months)** |  |  |  |  |  |  |  |  |  |  |  |  |  |  |
| 0-11 | 1868 | 302 | Ref |  | Ref |  | Ref |  | Ref |  | Ref |  | Ref |  |
| 12-23 | 1474 | 460 | 1.93 (1.53, 2.45) | < 0.001 | 1.88 (1.32, 2.67) | < 0.001 | 1.77 (1.27, 2.47) | 0.001 | 1.78 (1.28, 2.48) | 0.001 | 1.78 (1.28, 2.48) | 0.001 | 1.78 (1.28, 2.49) | 0.001 |
| 24-35 | 1344 | 481 | 2.22 (1.74, 2.83) | < 0.001 | 1.95 (1.01, 3.73) | 0.048 | 2.08 (1.51, 2.86) | < 0.001 | 2.08 (1.51, 2.86) | < 0.001 | 2.08 (1.51, 2.87) | < 0.001 | 2.08 (1.51, 2.87) | < 0.001 |
| 36-47 | 1371 | 486 | 2.19 (1.76, 2.74) | < 0.001 | 0.68 (0.10, 4.63) | 0.695 | 2.20 (1.59, 3.04) | < 0.001 | 2.21 (1.60, 3.06) | < 0.001 | 2.21 (1.60, 3.06) | < 0.001 | 2.22 (1.60, 3.07) | < 0.001 |
| 48-59 | 1381 | 585 | 2.62 (2.05, 3.36) | < 0.001 | 0.94 (0.14, 6.49) | 0.947 | 2.95 (2.10, 4.13) | < 0.001 | 2.96 (2.11, 4.15) | < 0.001 | 2.95 (2.10, 4.15) | < 0.001 | 2.97 (2.11, 4.17) | < 0.001 |
| **Child anaemia status** |  |  |  |  |  |  |  |  |  |  |  |  |  |  |
| Sever | 142 | 119 | Ref |  | Ref |  | Ref |  | Ref |  | Ref |  | Ref |  |
| Moderate | 3337 | 1260 | 0.45 (0.30, 0.67) | < 0.001 | 0.54 (0.34, 0.84) | 0.007 | 0.55 (0.34, 0.86) | 0.010 | 0.54 (0.34, 0.85) | 0.010 | 0.55 (0.35, 0.86) | 0.010 | 0.54 (0.35, 0.85) | 0.008 |
| Not anaemic | 2822 | 766 | 0.32 (0.21, 0.49) | < 0.001 | 0.33 (0.21, 0.52) | < 0.001 | 0.33 (0.21, 0.54) | < 0.001 | 0.33 (0.21, 0.53) | < 0.001 | 0.33 (0.21, 0.54) | < 0.001 | 0.33 (0.21, 0.53) | < 0.001 |
| **Size of child at birth** |  |  |  |  |  |  |  |  |  |  |  |  |  |  |
| Larger than average | 2485 | 565 | Ref |  | Ref |  | Ref |  | Ref |  | Ref |  | Ref |  |
| Average | 3243 | 931 | 1.26 (1.06, 1.50) | 0.01 | 1.27 (1.06, 1.53) | 0.010 | 1.27 (1.06, 1.53) | 0.010 | 1.27 (1.06, 1.52) | 0.010 | 1.27 (1.06, 1.52) | 0.011 | 1.27 (1.06, 1.52) | 0.009 |
| Smaller than average | 1711 | 819 | 2.10 (1.71, 2.59) | < 0.001 | 1.27 (1.06, 2.46) | < 0.001 | 1.97 (1.58, 2.45) | < 0.001 | 1.96 (1.58, 2.44) | < 0.001 | 1.96 (1.58, 2.44) | < 0.001 | 1.96 (1.58, 2.44) | < 0.001 |
| **Birthweight of child** |  |  |  |  |  |  |  |  |  |  |  |  |  |  |
| >= 2500 grams | 1031 | 148 | Ref |  | Ref |  | Ref |  | Ref |  | Ref |  | Ref |  |
| < 2500 grams | 118 | 60 | 3.52 (2.03, 6.10) | < 0.001 | 2.39 (1.26, 4.53) | 0.028 | 2.39 (1.27, 4.48) | 0.018 | 2.43 (1.30, 4.52) | 0.016 | 2.41 (1.29, 4.51) | 0.017 | 2.43 (1.31, 4.52) | 0.015 |
| **Diarrhoea in 2 weeks** |  |  |  |  |  |  |  |  |  |  |  |  |  |  |
| Yes | 825 | 361 | 1.48 (1.18, 1.86) | < 0.001 | 1.63 (1.27, 2.09) | 0.001 | 1.61 (1.26, 2.07) | 0.001 | 1.61 (1.26, 2.07) | 0.001 | 1.62 (1.26, 2.08) | 0.001 | 1.61 (1.26, 2.07) | 0.001 |
| No | 6600 | 1949 | Ref |  | Ref |  | Ref |  | Ref |  | Ref |  | Ref |  |
| **Maternal BMI** |  |  |  |  |  |  |  |  |  |  |  |  |  |  |
| Underweight | 1205 | 526 | 3.44 (2.34, 5.06) | < 0.001 | 2.52 (1.67, 3.80) | < 0.001 | 2.55 (1.68, 3.86) | < 0.0001 | 2.54 (1.68, 3.84) | < 0.001 | 2.53 (1.67, 3.83) | < 0.001 | 2.51 (1.67, 3.79) | < 0.001 |
| Normal | 5594 | 1703 | 2.40 (1.68, 3.43) | < 0.001 | 1.84 (1.27, 2.68) | 0.002 | 1.88 (1.29, 2.75) | 0.001 | 1.87 (1.28, 2.74) | 0.001 | 1.86 (1.27, 2.72) | 0.002 | 1.85 (1.27, 2.70) | 0.001 |
| Overweight | 559 | 71 | Ref |  | Ref |  | Ref |  | Ref |  | Ref |  | Ref |  |
| **Minimum food groups consumed in 24hrs** |  |  |  |  |  |  |  |  |  |  |  |  |  |  |
| < 4 food groups | 2269 | 662 | 1.48 (0.74, 2.97) | 0.077 | 1.82 (0.77, 4.31) | 0.346 | 1.84 (0.79, 4.26) | 0.237 | 1.79 (0.79, 4.08) | 0.235 | 1.77 (0.78, 4.02) | 0.245 | 1.79 (0.79, 4.09) | 0.236 |
| >= 4 food groups | 110 | 22 | Ref |  | Ref |  | Ref |  | Ref |  | Ref |  | Ref |  |

AOR, adjusted odds ratio; ANC, antenatal care; BMI, body mass index; COR, crude odds ratio; Ref, reference group; SNNP, southern nations, nationalities and people; WASH, water, sanitation and handwashing; Model 0, results from unadjusted univariable analysis; Model 1, adjusted for all variables with p value < 0.25 in model 0; Model 2, adjusted for water plus all variables with p value < 0.05 in Model 1; Model 3, adjusted for sanitation plus all variables with p value < 0.05 in Model 1; Model 4, adjusted for handwashing plus all variables with p value < 0.05 in Model 1; Model 5, adjusted for combined WASH facilities plus all variables with p value < 0.05 in Model 1.
