# Supplementary material for: The effect of access to water, sanitation and handwashing facilities on child growth indicators: Evidence from the Ethiopia Demographic and Health Survey 2016
Source: PLoS One. 2020 Sep 22;15(9):e0239313. doi: 10.1371/journal.pone.0239313 (PMC7508389; doi:10.1371/journal.pone.0239313)
Supplement: S3 Table — (DOCX) [file pone.0239313.s003.docx]

**S3 Table.** Univariable and multivariable analyses results of wasting for all confounding variables, EDHS 2016 (weighted n = 9607)

| **Variables** | **Wasting** | | **Model 0** | | **Model 1** | | **Model 2** | | **Model 3** | | **Model 4** | | **Model 5** | |
| --- | --- | --- | --- | --- | --- | --- | --- | --- | --- | --- | --- | --- | --- | --- |
|  | **No** | **Yes** | **COR (95%CI)** | **P value** | **AOR (95%CI)** | **P value** | **AOR (95%CI)** | **P value** | **AOR (95%CI)** | **P value** | **AOR (95%CI)** | **P value** | **AOR (95%CI)** | **P value** |
| **Water facility** |  |  |  |  |  |  |  |  |  |  |  |  |  |  |
| Improved | 4842 | 507 | 0.86 (0.68, 1.08) | 0.051 |  |  | 1.03 (0.82, 1.29) | 0.800 |  |  |  |  |  |  |
| Unimproved | 3694 | 452 | Ref |  |  |  | Ref |  |  |  |  |  |  |  |
| **Sanitation facility** |  |  |  |  |  |  |  |  |  |  |  |  |  |  |
| Improved | 836 | 86 | 0.91 (0.69, 1.21) | 0.357 |  |  |  |  | 1.07 (0.79, 1.46) | 0.649 |  |  |  |  |
| Unimproved | 7700 | 873 | Ref |  |  |  |  |  | Ref |  |  |  |  |  |
| **Handwashing facility** |  |  |  |  |  |  |  |  |  |  |  |  |  |  |
| Improved | 4531 | 484 | 0.90 (0.73, 1.12) | 0.155 |  |  |  |  |  |  | 1.05 (0.84, 1.31) | 0.665 |  |  |
| Unimproved | 4005 | 475 | Ref |  |  |  |  |  |  |  | Ref |  |  |  |
| **WASH facilities** |  |  |  |  |  |  |  |  |  |  |  |  |  |  |
| Improved | 486 | 40 | 0.72 (0.47, 1.10) | 0.127 |  |  |  |  |  |  |  |  | 1.13 (0.73, 1.75) | 0.588 |
| Unimproved | 8050 | 920 | Ref |  |  |  |  |  |  |  |  |  | Ref |  |
| **Region** |  |  |  |  |  |  |  |  |  |  |  |  |  |  |
| Addis Ababa | 202 | 8 | Ref |  | Ref |  | Ref |  | Ref |  | Ref |  | Ref |  |
| Tigray | 574 | 74 | 3.45 (2.01, 5.91) | < 0.001 | 2,17 (1.18, 4.00) | 0.013 | 2.06 (1.11, 3.80) | 0.022 | 2.13 (1.15, 3.94) | 0.017 | 2.13 (1.15, 3.94) | 0.019 | 2.19 (1.16, 4.12) | 0.016 |
| Afar | 76 | 17 | 5.97 (3.39, 10.52) | < 0.001 | 3,46 (1,75, 6.86) | 0.0004 | 3.01 (1.55, 5.85) | 0.001 | 3.10 (1.60, 6.01) | 0.001 | 3.10 (1.60, 6.01) | 0.001 | 3.19 (1.61, 6.32) | 0.001 |
| Amhara | 1684 | 187 | 2.97 (1.72, 5.16) | < 0.001 | 2.06 (1.06, 4.00) | 0.032 | 1.84 (0.96, 3.53) | 0.066 | 1.90 (0.99, 3.65) | 0.053 | 1.90 (0.99, 3.65) | 0.070 | 1.96 (1.00, 3.83) | 0.051 |
| Oromia | 3774 | 447 | 3.18 (1.87, 5.41) | < 0.0001 | 2.17 (1.14, 4.10) | 0.018 | 2.04 (1.09, 3.80) | 0.026 | 2.10 (1.12, 3.96) | 0.022 | 2.10 (1.12, 3.96) | 0.025 | 2.16 (1.12, 4.18) | 0.022 |
| Somali | 313 | 94 | 8.07 (4.54, 14.35) | < 0.001 | 5.54 (2.79, 10.99) | < 0.001 | 4.86 (2.52, 9.38) | < 0.001 | 4.93 (2.57, 9.45) | < 0.001 | 4.93 (2.57, 9.45) | < 0.001 | 5.13 (2.57, 10.24) | < 0.001 |
| Benishangul-Gumuz | 90 | 11 | 3.25 (1.79, 5.90) | < 0.001 | 2.44 (1.22, 4.86) | 0.012 | 2.09 (1.05, 4.15) | 0.036 | 2.18 (1.10, 4.33) | 0.026 | 2.18 (1.10, 4.33) | 0.032 | 2.24 (1.20, 4.55) | 0.027 |
| SNNP | 1853 | 123 | 1.78 (1.01, 3.15) | 0.0469 | 1.42 (0.74, 2.72) | 0.296 | 1.21 (0.63, 2.32) | 0.559 | 1.25 (0.65, 2.40) | 0.502 | 1.25 (0.65, 2.40) | 0.546 | 1.29 (0.66, 2.52) | 0.461 |
| Gambela | 19 | 3 | 4.35 (2.38, 7.95) | < 0.001 | 3.02 (1.58, 5.79) | 0.001 | 2.80 (1.47, 5.36) | 0.002 | 2.91 (1.51, 5.59) | 0.001 | 2.91 (1.51, 5.59) | 0.002 | 2.99 (1.52, 5.87) | 0.002 |
| Harari | 18 | 2 | 3.30 (1.76, 6.17) | < 0.001 | 2.48 (1.24, 4.95) | 0.011 | 2.35 (1.18, 4.66) | 0.015 | 2.41 (1.21, 4.78) | 0.012 | 2.41 (1.21, 4.78) | 0.014 | 2.49 (1.23, 5.06) | 0.012 |
| Dire Dawa | 33 | 4 | 3.12 (1.69, 5.75) | < 0.001 | 2.17 (1.11, 4.23) | 0.023 | 2.03 (1.05, 3.91) | 0.034 | 2.07 (1.08, 3.97) | 0.030 | 2.07 (1.08, 3.97) | 0.030 | 2.13 (1.09, 4.16) | 0.027 |
| **Household head** |  |  |  |  |  |  |  |  |  |  |  |  |  |  |
| Male | 7449 | 856 | Ref |  | Ref |  | Ref |  | Ref |  | Ref |  | Ref |  |
| Female | 1188 | 113 | 0.83 (0.63, 1.09) | 0.073 | 0.69 (0.51, 0.95) | 0.021 | 0.76 (0.57, 1.01) | 0.060 | 0.76 (0.57, 1.01) | 0.062 | 0.76 (0.57, 1.01) | 0.062 | 0.76 (0.57, 1.01) | 0.062 |
| **Wealth index** |  |  |  |  |  |  |  |  |  |  |  |  |  |  |
| Poorest | 1923 | 312 | Ref |  | Ref |  | Ref |  | Ref |  | Ref |  | Ref |  |
| Poorer | 2037 | 221 | 0.67 (0.49, 0.92) | 0.012 | 0.81 (0.56, 1.18) | 0.273 | 0.80 (0.55, 1.15) | 0.229 | 0.80 (0.55, 1.16) | 0.238 | 0.80 (0.55, 1.16) | 0.232 | 0.80 (0.55, 1.16) | 0.238 |
| Middle | 1809 | 210 | 0.72 (0.52, 0.99) | 0.043 | 0.89 (0.62, 1,28) | 0.517 | 0.87 (0.62, 1,22) | 0.419 | 0.87 (0.61, 1,24) | 0.442 | 0.87 (0.61, 1,24) | 0.447 | 0.87 (0.61, 1,24) | 0.451 |
| Richer | 1610 | 121 | 0.46 (0.32, 0.67) | < 0.001 | 0.59 (0.39, 0.88) | 0.012 | 0.58 (0.39, 0.86) | 0.006 | 0.58 (0.39, 0.87) | 0.009 | 0.58 (0.39, 0.86) | 0.007 | 0.58 (0.39, 0.87) | 0.008 |
| Richest | 1258 | 106 | 0.52 (0.36, 0.75) | 0.001 | 0.80 (0.50, 1.27) | 0.339 | 0.79 (0.51, 1.23) | 0.294 | 0.79 (0.50, 1.24) | 0.306 | 0.79 (0.51, 1.22) | 0.288 | 0.78 (0.50, 1.22) | 0.282 |
| **Childbirth order** | 8637 | 970 | 1.06 (1.02, 1.10) | < 0.001 | 1.05 (1.00, 1.10) | 0.038 | 1.05 (1.00, 1.09) | 0.030 | 1.05 (1.00, 1.09) | 0.031 | 1.05 (1.01, 1.09) | 0.030 | 1.05 (1.01, 1.09) | 0.030 |
| **Size of child at birth** |  |  |  |  |  |  |  |  |  |  |  |  |  |  |
| Larger than average | 2769 | 251 | Ref |  | Ref |  | Ref |  | Ref |  | Ref |  | Ref |  |
| Average | 3707 | 402 | 1.20 (0.93, 1.52) | 0.151 | 1.20 (0.94, 1.54) | 0.153 | 1.19 (0.93, 1.52) | 0.160 | 1.19 (0.93, 1.52) | 0.158 | 1.19 (0.94, 1.52) | 0.156 | 1.19 (0.94, 1.52) | 0.156 |
| Smaller than average | 2160 | 316 | 1.61 (1.25, 2.08) | < 0.001 | 1.46 (1.13, 1.90) | 0.004 | 1.53 (1.19, 1.98) | 0.001 | 1.54 (1.19, 1.98) | 0.001 | 1.54 (1.19, 1.99) | 0.001 | 1.54 (1.19, 1.99) | 0.001 |
| **Maternal BMI** |  |  |  |  |  |  |  |  |  |  |  |  |  |  |
| Underweight | 1466 | 241 | 2.98 (1.58, 5.59) | 0.001 | 2.80 (1.38, 5.67) | 0.004 | 2.73 (1.36, 5.49) | 0.005 | 2.74 (1.36, 5.53) | 0.005 | 2.73 (1.35, 5.52) | 0.005 | 2.75 (1.36, 5.58) | 0.005 |
| Normal | 6499 | 684 | 1.91 (1.07, 3.41) | 0.030 | 1.97 (1.04, 3.75) | 0.038 | 1.95 (1.02, 3.71) | 0.043 | 1.96 (1.03, 3.73) | 0.042 | 1.95 (1.02, 3.72) | 0.043 | 1.96 (1.03, 3.76) | 0.042 |
| Overweight | 590 | 33 | Ref |  | Ref |  | Ref |  | Ref |  | Ref |  | Ref |  |

AOR, adjusted odds ratio; BMI, body mass index; COR, crude odds ratio; Ref, reference group; SNNP, southern nations, nationalities and people; WASH, water, sanitation and handwashing; Model 0, results from unadjusted univariable analysis; Model 1, adjusted for all variables with p value < 0.25 in model 0; Model 2, adjusted for water plus all variables with p value < 0.05 in Model 1; Model 3, adjusted for sanitation plus all variables with p value < 0.05 in Model 1; Model 4, adjusted for handwashing plus all variables with p value < 0.05 in Model 1; Model 5, adjusted for combined WASH facilities plus all variables with p value < 0.05 in Model 1.
